# Simultaneous Copy Number Losses within Multiple Subtelomeric Regions in Early-Onset Type2 Diabetes Mellitus

**DOI:** 10.1371/journal.pone.0088602

**Published:** 2014-04-07

**Authors:** Shinjiro Kodama, Tetsuya Yamada, Junta Imai, Shojiro Sawada, Kei Takahashi, Sohei Tsukita, Keizo Kaneko, Kenji Uno, Yasushi Ishigaki, Yoshitomo Oka, Hideki Katagiri

**Affiliations:** 1 Division of Metabolism and Diabetes, Tohoku University Graduate School of Medicine, Sendai, Japan; 2 Japan Science and Technology Agency, CREST, Tokyo, Japan; Institut Jacques Monod, France

## Abstract

Genetic factors play very important roles in the onset and progression of type 2 diabetes mellitus (T2DM). However, the genetic factors correlating with T2DM onset have not as yet been fully clarified. We previously found that copy number losses in the subtelomeric region on chromosome 4p16.3 were detected in early-onset Japanese T2DM patients (onset age <35 years) at a high frequency. Herein, we additionally found two novel copy number losses within the subtelomeric regions on chromosomes 16q24.2-3 and 22q13.31-33, which have significant associations with early-onset Japanese T2DM. The associations were statistically significant by Fisher's exact tests with *P* values of 5.19×10^−3^ and 1.81×10^−3^ and odds ratios of 5.7 and 4.4 for 16q24.2-3 and 22q13.31-33, respectively. Furthermore, copy number variation (CNV) analysis of the whole genome using the CNV BeadChip system verified simultaneous copy number losses in all three subtelomeric regions in 11 of our 100 T2DM subjects, while none of 100 non-diabetic controls showed the copy number losses in all three regions. Our results suggest that the mechanism underlying induction of CNVs is involved in the pathogenesis of early-onset T2DM. Thus, copy number losses within multiple subtelomeric regions are strongly associated with early-onset T2DM and examination of simultaneous CNVs in these three regions may lead to the development of an accurate and selective procedure for detecting genetic susceptibility to T2DM.

## Introduction

Numbers of patients with type 2 diabetes mellitus (T2DM) have been increasing annually worldwide. Environmental factors, such as aging, westernized high-fat diets, lack of exercise and everyday stress affect the onset and progression of T2DM. On the other hand, according to epidemiological studies, genetic factors also play a major role [1]. For example, concordance rates and heritability for T2DM are higher in identical than in fraternal twins [2]. When both parents have T2DM, the risk of this disease in their children is three to four times higher than that of the offspring of non-diabetics [3]. Furthermore, the sibling recurrence-risk ratios for T2DM were substantially elevated in families with diabetes in both a parent and a grandparent [4]. Therefore, many investigators worldwide have been intensively searching for genetic factors associated with susceptibility to T2DM.

Loci for rare monogenic forms of familial diabetes, such as maturity-onset diabetes of the young (MODY) [5], including glucokinase gene mutations [6], mitochondrial diabetes [7], [8], and Wolfram syndrome [9], have been demonstrated in small proportions of patients. However, the genetic etiology of familial T2DM has not been revealed in the majority of cases. To identify genetic factors conferring susceptibility to T2DM, genome-wide association studies (GWASs) using single nucleotide polymorphism (SNP) markers have been performed. These GWASs and replication studies have identified multiple susceptibility loci, such as *TCF7L2* [10], *GLIS3* [11], *KCNK16* [11], [12], *MAEA* [12] and *KCNQ1* [13], [14]. However, their odds ratios are consistently in the 1.1–1.4 range [9], [13], suggesting their contributions to be relatively low. Furthermore, among the loci detected to date, the initially-reported associations have not necessarily been replicated in subsequent studies [15], [16].

Other chromosomal mutations, which cover wider genomic regions, include duplication, deletion, insertion, translocation, block substation, indel (insertion-deletion) and copy number variations (CNVs). Among these genetic variations, CNVs are increasingly being recognized due to their major contributions to a number of diseases, including certain malignancies. To date, the existence of CNV was considered to be very rare, since CNV would have a fatal impact on brain function and/or the survival of individuals [17]. However, recent advances in human genome studies have totally revised this concept. Many case reports have made it clear that CNVs exist even in healthy individuals [15], [16]. For example, a CNV in the CCR3L1 region is reportedly related to HIV infection [18].A CNV in the FCGR3 gene was reported to correlate with glomerulonephritis in systemic lupus erythematosus patients [19], [20]. Therefore, CNVs are recognized as significant genetic contributors to human diseases [21]. Furthermore, in common diseases, such as schizophrenia [22], [23], autism [24]–[27] and bipolar disorder [28], CNVs have been demonstrated to be contributory genetic factors. However, there are only a few reports demonstrating associations between CNV and T2DM [29]–[32]. Recently, we demonstrated copy number losses within the 1.3-Mb subtelomeric region on chromosome 4p16.3 in a substantial portion of early-onset Japanese T2DM subjects [32].

In our search for other CNVs conferring genetic susceptibility to T2DM, we performed genome-wide CNV analyses in early-onset Japanese T2DM subjects. Early-onset patients had developed T2DM before environmental and acquired factors would have had major impacts, suggesting these patients to have stronger inherited factors. Therefore, we enrolled T2DM subjects whose onsets had been before 35 years of age. As a result, 94 of our 100 T2DM subjects had a family history of T2DM within third-degree relatives [32]. We first screened CNVs in the whole genome using the deCODE-Illumina CNV370K BeadChip system which focuses on the CNV-rich region of the human genome, followed by validation and characterization using an Agilent region-targeted high-density custom-made oligonucleotide tiling microarray [32]. We found two novel copy number losses within the multiple subtelomeric regions on chromosomes 16q24.2-3 and 22q13.31-33, which have associations with early-onset diabetes. In addition, importantly, copy number losses in chromosomes 4p16.3, 16q24.2-3 and 22q13.31-33 were often detected simultaneously in the same subjects. In particular, all three copy number losses were verified in 11 of our 100 T2DM patients, while none of the non-diabetic controls showed simultaneous copy number losses in these regions. Our results suggest that the combination of CNVs in these three genome regions is a potential DNA marker for T2DM, and that the mechanism underlying induction of CNVs, rather than a single gene in these CNV regions, is involved in the pathogenesis of T2DM.

## Materials and Methods

### Study population

We considered early onset of T2DM to reflect the presence more of genetic than of environmental factors. Therefore, we enrolled diabetic patients with early-onset disease as study subjects. One hundred unrelated Japanese T2DM patients, who had developed T2DM before 35 years of age and whose maximum body mass index (BMI) was less than 35 (kg/m^2^), were the same subjects as in our previous study [32]. They were recruited at Tohoku University Hospital and affiliated hospitals and medical clinics. Diabetes had been diagnosed by applying the WHO criteria. Type 1 diabetes mellitus was excluded based on clinical features and the absence of anti-GAD (glutamic acid decarboxylase) antibodies or anti-IA-2 (insulinoma-associated antigen-2) antibodies. Patients with diabetes mellitus due to hepatic disease, pancreatic disease, other endocrine disorders or mitochondrial DNA mutations, and those with drug-induced diabetes, based on laboratory data and clinical history, were excluded. We also studied 100 non-diabetic control subjects, again the same subjects as in our previous study [32]. These control subjects were enrolled using the following criteria: 60 or more years of age, no prior diagnosis of diabetes mellitus, HbA1c less than 6.4% and absence of a family history of T2DM within third-degree relatives. Applying these criteria allowed us to exclude subjects with a high likelihood of later developing T2DM. HbA1c (%) was estimated as an NGSP (National Glycohemoglobin Standardization Program) equivalent value (%) calculated by the following formula: HbA1c (%) = HbA1c (JDS: Japan Diabetes Society value) (%)+0.4%. The value of HbA1c (JDS) (%) measured based on the previous Japanese standard substance and measurement methods.

Genetic analysis of human subjects was approved by the ethics committee of Tohoku University Graduate School of Medicine. After a detailed description of study participation, written informed consent was obtained for each subject.

### Screening methods with the Whole-Genome CNV BeadChip System

As previously reported [32], we screened the whole genome by CNV analysis using the deCODE-Illumina CNV370K BeadChip system (Illumina Infinium System, deCODE Genetics, Inc., Iceland), which, in addition to Hap300 SNP marker contents, has CNV probes designed to target the CNV-rich region of the whole genome. The CNV portion of the platform consists of probes covering CNV rich regions of the genome, such as megasatellites (tandem repeats >500 bp), duplicons (regions flanked by highly homologous segmental duplication >1 kb), unSNPable regions (>15 kb gaps in HapMap SNP map, and 5–15 kb gaps with >2SNPs with Hardy-Weinberg failure), and CNVs registered in the Database of Genomic Variants. The CNV portion of the probe content consists of 15,559 CNV segments covering 190 Mb, or 6% of the human genome. The platform has been tested in 4000 Icelandic and HapMap samples.

Data analysis of the deCODE-Illumina CNV chip was carried out using DosageMiner software developed by deCODE genetics, and loss/gain analysis consisted of the following four steps; (1) intensity normalization and GC content correction, (2) removal of batch effects using principal component analysis, (3) calling of clusters using a Gaussian mixture model, and (4) determination of CNV type using graphical constraints. In brief, CNVs were identified when CNV events stood out in the data, as all sample intensities for CNV probes should be increased or decreased relative to neighboring probes that are not in a CNV region. To determine deviations in signal intensity we started by normalizing the intensities. The normalized intensities for each color channel were determined by an equation and fit formula developed by deCODE genetics. A stretch with occurrence of more than one marker showing an abnormality in the copy number in a consecutive stretch in the genome is considered more likely to be evidence of deletion or gain [22].

### High-Density Custom-Made Oligonucleotide Tiling Microarray Analysis

DNA samples from early-onset T2DM subjects were subjected to Agilent's high-density custom-made oligonucleotide tiling microarray analysis based on an array comparative genomic hybridization (aCGH) assay as previously reported. We fabricated a custom-designed microarray targeted to a 1.75-Mb genome region in the subtelomere at 16q24.2-3 (Chr. 16: 86,950,000–88,700,000 (NCBI Build 36.1, hg18)), and a 4.8-Mb genome region in the subtelomere at 22q13.31-33 (Chr. 22: 44,750,000–49,550,000 (NCBI Build 36.1, hg18)) according to previously described methods [33], [34]. In short, we used the Agilent website (http://earray.chem.agilent.com/earray/) to select and design our custom tiling array; the array consisted of probes 60-mer in size (Agilent Technologies, Santa Clara, CA, USA).

Tiling-aCGH experiments were performed essentially as described previously [35]. In brief, test and reference (NA19000, a Japanese male from the HapMap project) genomic DNAs (250 ng per sample) were fluorescently labeled with Cy5 (test) and Cy3 (reference) with a ULS Labeling Kit (Agilent Technologies).

For each sample, respective labeling reactions were mixed and then separated prior to hybridization for each of the arrays. Labeled test and reference DNAs were combined, denatured, pre-annealed with Cot-1 DNA (Invitrogen, 1600 Faraday Avenue, Carlsbad, CA, USA) and a blocking agent, and then hybridized to the arrays for 24 hours in a rotating oven at 65°C and 20 rpm (Agilent Technologies). After hybridization and washing, the arrays were scanned at 3 µm resolution with an Agilent G2505C scanner. Images were analyzed with Feature Extraction Software 10.7.3.1 (Agilent Technologies), with the CGH 107 Sep09 protocol for background subtraction and normalization. Abnormal copy numbers, losses, and gains, in a complex multi-copy variable region by high-density tiling array were detected by deviation of probe log2 ratios that exceeded a threshold of 1 SD from the median probe ratio, according to procedures described previously [35]–[37]. We defined two copy number classes, that is, “unchanged copy number” and “copy number loss.” “Unchanged copy number” was defined as the log2 ratio staying within the mean± 1 SD distribution for the normal population. “Copy number loss” was considered to be present when the downward-deviation of log2 ratios exceeded a threshold of 1 SD from the median probe ratio.

### Statistical analysis

Statistical analyses were performed using JMP and Microsoft excel. The Chi square tests were used to compare characteristics of cases and controls, and the clinical characteristics of T2DM associated with combined copy number loss. Fisher's exact tests were used to examine whether there were differences in the copy number losses between cases and controls.

## Results

In searching for other CNVs associated with early-onset T2DM, we screened the whole genome for CNVs using the deCODE-Illumina CNV370K BeadChip system in the same 100 early-onset Japanese T2DM subjects and 100 non-diabetic controls that we reported previously [32]. Characteristics of the diabetic cases and non-diabetic controls are shown in Table 1. To search for other CNVs as genetic variations possibly conferring susceptibility to T2DM, we performed genome-wide CNV analyses in early-onset Japanese T2DM subjects and non-diabetic controls. Early-onset patients had developed T2DM before environmental factors would have had major impacts, suggesting these patients to have stronger genetic factors. Therefore, we selected 100 Japanese T2DM subjects whose onsets had been before 35 years of age. In fact, 94 of our 100 T2DM subjects had a family history of T2DM within third-degree relatives (Table 1). As non-diabetic controls, genomes obtained from subjects who were at least 60 years of age and had not developed diabetes were used. In addition to the CNV in 4p16.3 [32], interestingly, those of 16q24.2-3 and 22q13.31-33 were found to be located on the subtelomeric regions in each chromosome. At the CNV on 16q24.2-3, 15 of the 100 T2DM subjects displayed copy number loss around this CNV marker, compared with only 3 of the 100 non-diabetic control samples. At the CNV on 22q13.31-33, 22 of the 100 T2DM subjects displayed copy number loss around this CNV marker, compared with only 6 of the 100 control samples. Hereafter, we focused on the two subtelomeric CNVs on 16q24.2-3 and 22q13.31-33.

**Table 1:** Characteristics of T2DM patients and controls.

	Cases (n = 100)	Controls (n = 100)	P
Male	59/100	35/100	0.0011
Age of onset	21.9±7.3	–	–
Family history of T2DM	94/100	0/100	0.0001
Age of examination	37.1±13.0	72.8±6.4	0.0001
Maximum body mass index (kg/m^2^)	28.0±4.2	25.2±3.2	0.64
HbA1c (NGSP)(%)	8.4±2.2	5.5±0.3	0.0008
Postprandial plasma glucose (mg/dl)	171.4±79.7	99.4±15.6	0.0006

**Figure 1:**
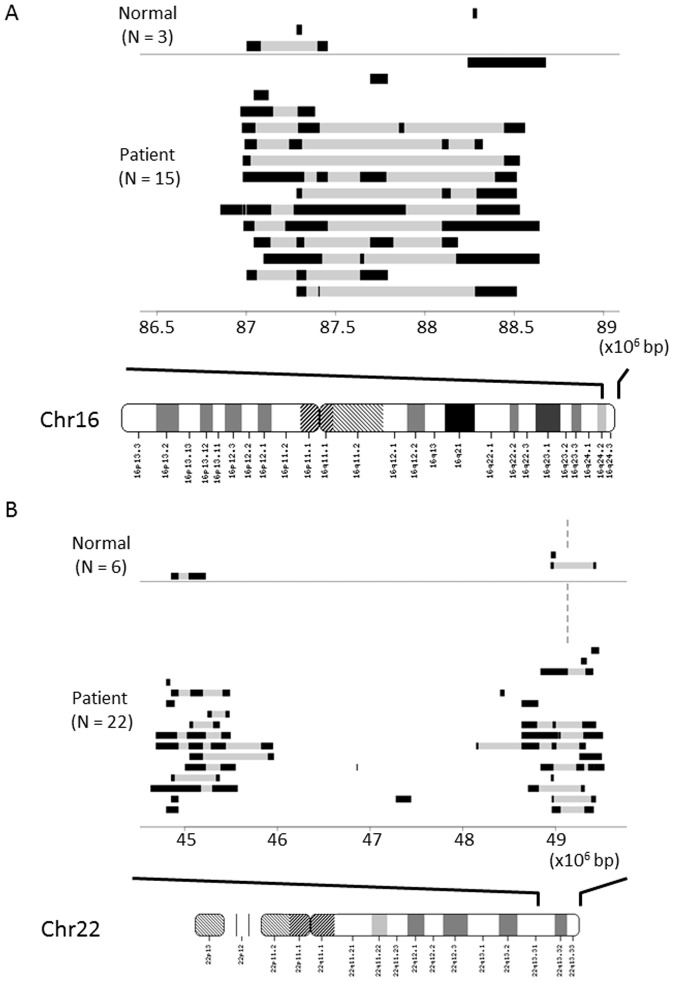
Genomic region harboring copy number loss of 1.75-Mb 16q24 (A) or 1.85-Mb 22q13 (B) subtelomeric region in 15 early-onset T2DM patients. A) Data obtained employing the deCODE/Illumina CNV370K chip system were analyzed using the PennCNV program. Genome structures of the 15 patients and 3 normal subjects are aligned as horizontal bars from genome position 86,950,000 (left) to position 88,700,000 (right). Dark solid horizontal bars represent the extent of copy number loss in each of the subjects. Gray regions between the dark bars represent intervals where copy number loss could not be inferred due to poor probe coverage. The lower map is an ideogram of chromosome 16. Position is given relative to NCBI Build 36 for chromosome 16. B) Data obtained employing the deCODE/Illumina CNV370K chip system were analyzed using the PennCNV program. Genome structures of the 22 patients and 6 normal subjects are aligned as horizontal bars from genome position 44,750,000 (left) to position 49,550,000 (right). Dark solid horizontal bars represent the extent of copy number loss in each of the subjects. Gray regions between the dark bars represent intervals where copy number loss could not be inferred due to poor probe coverage. The lower map is an ideogram of chromosome 22. Positions are given relative to NCBI Build 36 for chromosome 22.

Figure 1 shows the patterns of alterations in copy number loss observed in the 100 early-onset Japanese T2DM subjects and non-diabetic controls. Among diabetic subjects, 15 showed copy number losses in the 16q24.2-3 subtelomeric region, whereas only 3 of the 100 non-diabetic control samples had copy number losses in this region (*P* = 5.19×10−3, OR = 5.7, 95% confidence interval 1.6–20.4) (Figure 1A). For the 22q13.31-33 subtelomeric regions, 22 diabetic subjects showed copy number losses around the gap region, whereas only 6 of the 100 non-diabetic control samples had copy number losses in this region (*P* = 1.81×10−3, OR = 4.4, 95% confidence interval 1.7–11.4) (Figure 1B). We observed two copy number patterns, that is, “unchanged copy number” and “copy number loss”, at these two regions in our diabetic and control populations, while none of our subjects had detectable “copy number gain”. Copy number loss was frequently observed at the 16q24.2-3 and 22q13.31-33 subtelomeric regions in early-onset T2DM subjects.

**Figure 2:**
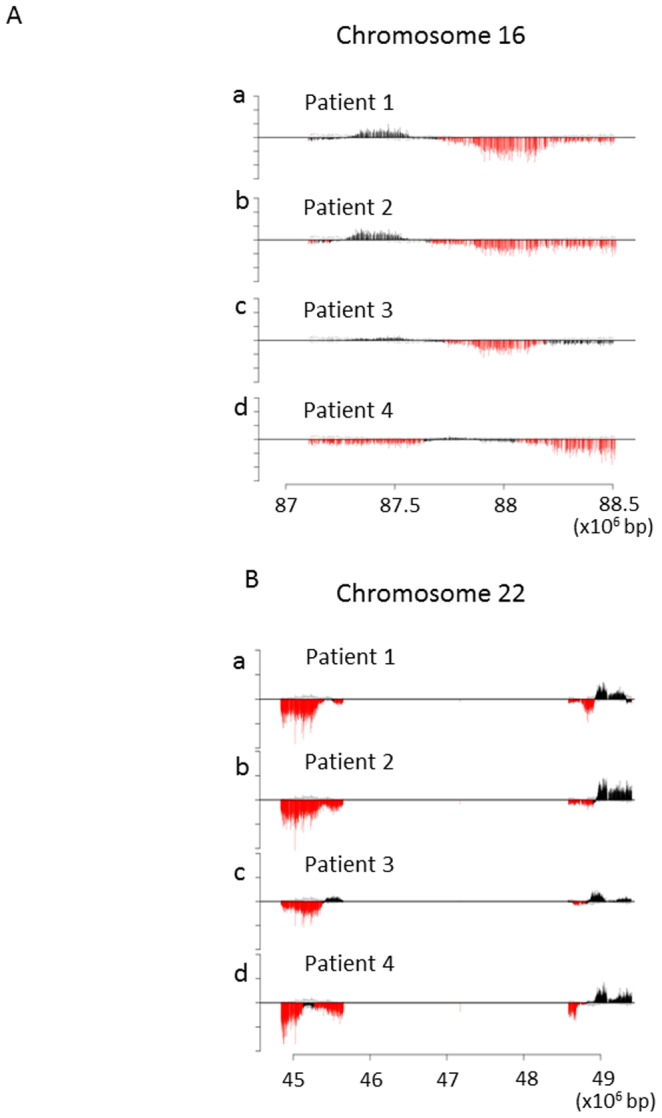
Structural details of copy number loss of 1.75-Mb 16q24 (A) or 1.85-Mb 22q13 (B) subtelomeric region resolved by high-density tiling microarray. A) The log2 ratio (y-axis) was plotted using the moving average along the genome position (x-axis). Representative early-onset T2DM data, from patients 1, 2, 3, and 4, respectively, are shown in Figures 2.1(a), 2.1(b), 2.1(c), and 2.1(d). The black vertical bar shows the copy number plot along the genome. The two pale lines indicate the normal range of average log2 ratios for probes among normal individuals. Copy number losses are displayed as red vertical bars. “Copy number loss” was considered to be present when the downward-deviation of log2 ratios exceeded a threshold of 1SD from the median probe ratio. B) The log2 ratio (y-axis) was plotted using the moving average along the genome position (x-axis). Representative early-onset T2DM data from patients 1, 2, 3, and 4, respectively, are shown as Figures 2.2(a), 2.2(b), 2.2(c), and 2.2(d). The black vertical bar shows the copy number plot along the genome. The two pale lines indicate the normal range of average log2 ratios for probes in normal individuals. Copy number losses are displayed as red vertical bars. “Copy number loss” was considered to be present when the downward-deviation of log2 ratios exceeded a threshold of 1SD from the median probe ratio.

**Figure 3:**
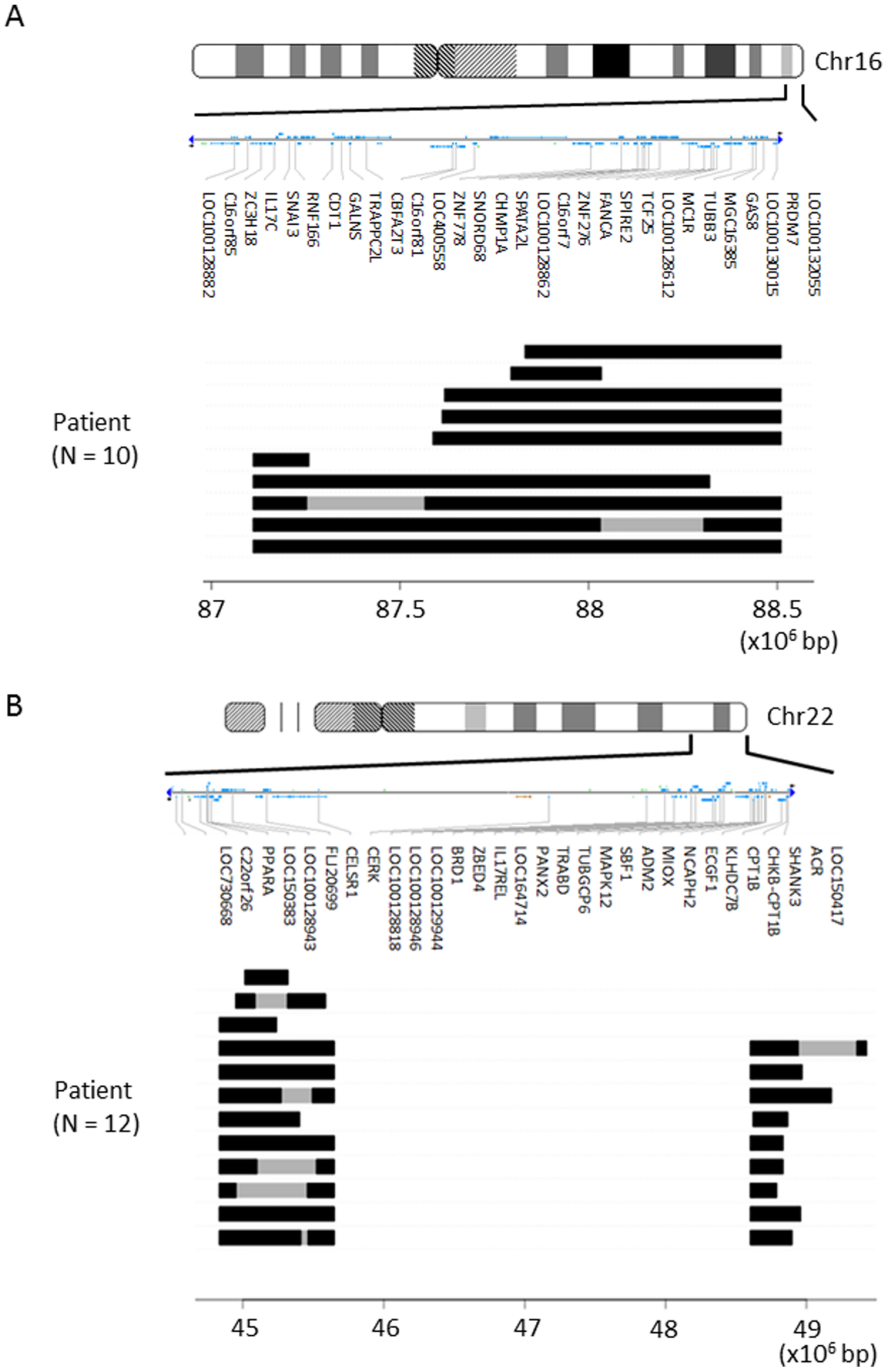
Extents of copy number losses within the 1.6-Mb 16q24 (A) and 4.8-Mb 22q13 (B) subtelomeric regions in early-onset T2DM patients as revealed by high-density oligonucleotide tiling microarray analysis. A) Dark horizontal bars represent the extents of copy number loss in each region in patients, as revealed by the Agilent custom tiling array analysis. Genome structures of the 10 patients subjects are aligned as horizontal bars from genome position 87,000,000 (left) to position 88,600,000 (right). Gray regions represent proximal ends of stretches where copy number status could not be inferred due to the presence of multiple low copy repeats. The upper map is an ideogram of chromosome 16 and the positions of putative genes in the 16q24 region described in the NCBI Map Viewer (http://www.ncbi.nlm.nih.gov/projects/mapview). Positions are given relative to NCBI Build 36 for chromosome 16. B) Dark horizontal bars represent the extent of copy number loss in each of the patients, as revealed by the Agilent custom tiling array analysis. Genome structures of the 12 patients subjects are aligned as horizontal bars from genome position 44,750,000 (left) to position 49,550,000 (right). Gray regions represent proximal ends of the stretch where copy number status could not be inferred due to the presence of multiple low copy repeats. The upper map is an ideogram of chromosome 22 and the positions of putative genes in the 22q13 region described in the NCBI Map Viewer (http://www.ncbi.nlm.nih.gov/projects/mapview). Positions are given relative to NCBI Build 36 for chromosome 22.

To verify the CNV BeadChip results, we analyzed copy number changes along these two regions in the subtelomere using a high-density custom-made oligonucleotide tiling microarray. Structural details of the copy number losses in each of four representative T2DM patients with copy number losses in 16q24.2-3 and 22q13.31-33 subtelomeric regions are shown in Figures 2A and 2B, respectively. Individual copy number plots using moving average (*y*-axis) versus distance along the chromosome (*x-*axis) are presented. For comparison, copy number plots of healthy individuals who did not exhibit copy number alterations in the region are also shown ((e) and (f) of Figures 2A and 2B). Using this procedure, we examined copy number losses in the 16q24.2-3 and 22q13.31-33 subtelomeric regions in 10 of 15 and 12 of 22 subjects, respectively, in whom copy number losses in each region had been detected by means of the first genome-wide screening using the deCODE-Illumina CNV370K BeadChip system. Copy number losses were confirmed in all 22 subjects examined (Figures 3A and 3B). In addition, the high-density tiling microarray further verified these copy number losses to actually be segmental losses. Thus, the first screening results were confirmed by the more detailed procedure.

**Figure 4:**
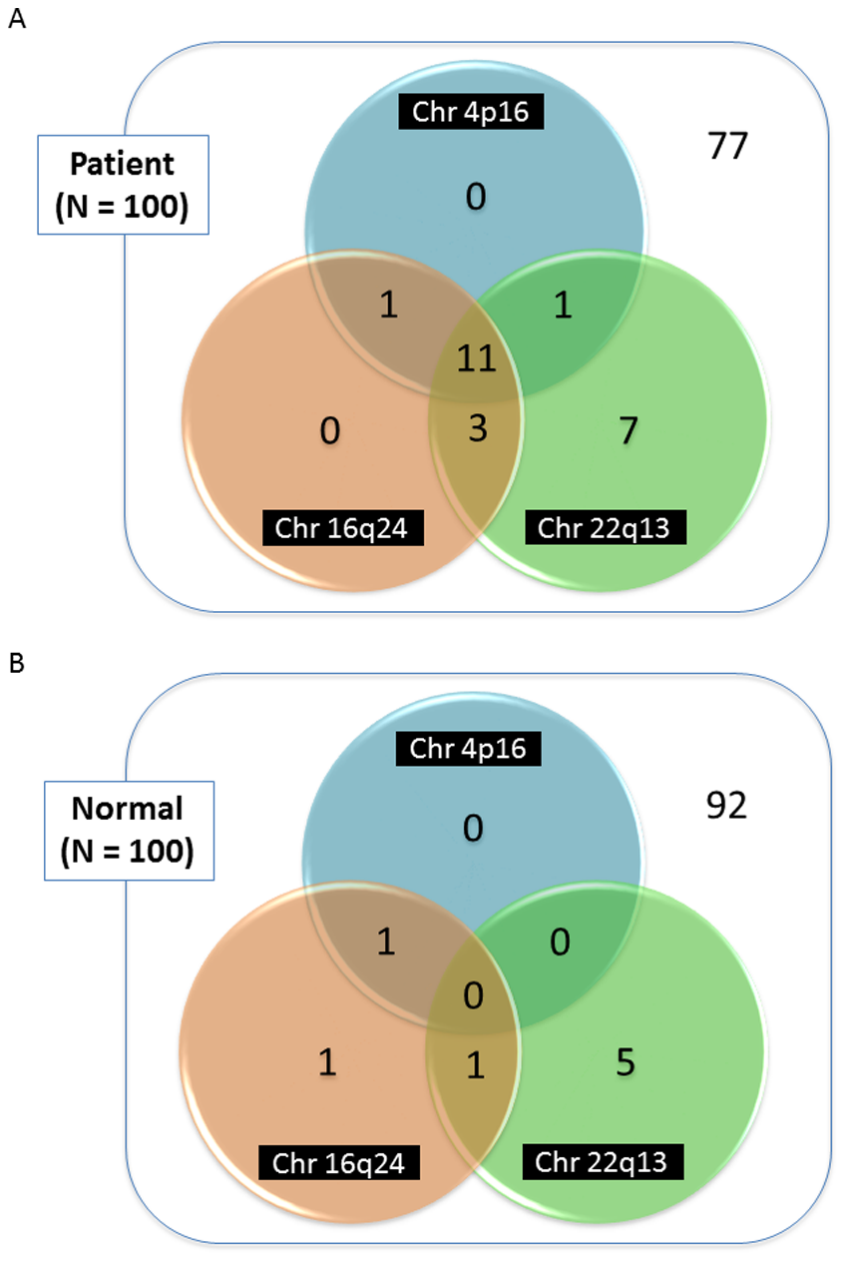
Details of the numbers of patients and controls with copy number loss in each region verified by the deCODE-Illumina CNV370K BeadChip system. Copy number losses were verified in 14 of the 100 T2DM patients in both 16q24.2-3 and 22q13.31-33, while only one of the 100 controls had copy number losses in these two regions. Copy number losses were verified in all three regions in 11 of the 100 T2DM patients, while none of the 100 controls had copy number losses in all three regions.

As we carried out these analyses, we recognized that the CNVs were often detected in the same subjects. For instance, all 10 subjects who were confirmed to have the copy number loss in 16q24.2-3 using the high-density tiling microarray analysis simultaneously had the copy number loss in 22q13.31-33. Therefore, we calculated the frequencies of these two CNVs as well as the one on 4p16.3 [32] in all 100 diabetic and 100 non-diabetic subjects, according to the results obtained using the deCODE-Illumina CNV BeadChip system. As shown in Figure 4A, all diabetic subjects with copy number loss on 4p16.3 or 16q24.2-3 had another CNV(s). In addition, 15 of the 22 diabetics with copy number loss on 22q13.31-33 simultaneously had another CNV(s). Intriguingly, 11 of the 100 T2DM subjects had copy number losses in all three subtelomeric regions (Figures 4 A), while none of the 100 controls had copy number losses in all three regions (Figure 4B) (*P* = 7.00×10^−4^ by Fisher's exact test). In contrast, of the study subjects with copy number losses in two of the three subtelomeric regions, 5 were diabetic (Figures 4 A), while 2 were controls (Figure 4B), with the difference not reaching statistical significance (*P* = 4.45×10^−1^). Thus, simultaneous copy number losses in these three different chromosomes are clearly associated with T2DM development and may become a novel DNA marker, usuful at least for detecting subjects likely to have early-onset T2DM.

We next examined the clinical characteristics of T2DM associated with combined copy number losses. Sex and onset age did not differ between patients with copy number losses in all three subtelomeric regions (n = 11) and those with no copy number losses in all three regions (n = 89). All 11 patients with copy number losses in all three subtelomeric regions had a family history of T2DM within third-degree relatives, supporting the notion of combined copy number losses being a genetic factor conferring high susceptibility to T2DM development. However, maximal body mass index, HbA1c and postprandial plasma glucose levels did not differ between these two groups (Table 2). In addition, urinary C-peptide reactivity levels and ratios of receiving insulin therapy were similar (Table 2), suggesting that patients with copy number losses in all three regions do not have specific clinical features. Therefore, it is possible that these combined copy number losses would often be detectable in early-onset T2DM patients. Thus, detection of these copy number losses may be a novel and useful diagnostic measure for predicting the early onset of T2DM.

**Table 2:** Characteristics of T2DM patients with and without CNVs in all three regions based on analysis using the deCODE-Illumina CNV BeadChip system.

	Patients with copy number losses in all three regions (n = 11)	Patients without copy number losses in all three regions (n = 89)	P
male	6/11	53/89	0.75
Age of onset	21.1±8.9	22.3±7.9	0.42
Family history of T2DM			
Yes	11/11	83/89	0.37
Maximum body mass index (kg/m^2^)	28.8±5.3	27.9±3.9	0.31
HbA1c (NGSP)(%)	8.3±2.8	8.4±2.3	0.54
Postprandial plasma glucose (mg/dl)	169.2±68.9	174.2±76.2	0.63
Use of insulin injection therapy			
Yes	9/11	54/89	0.74
Urine C-peptide reactivity level (µg/day)	67.8±88.3	59.9±91.2	0.29

Onset age, maximum body mass index, postprandial plasma glucose levels, family history, HbA1c levels, and urinary C-peptide reactivity levels did not differ between the 11 patients with copy number losses in all three regions and the other 89 patients.

## Discussion

This study revealed two novel copy number losses in the subtelomeric regions in 16q24.2-3 and 22q13.31-33. Our initial genome-wide screening with the deCODE-Illumina CNV370K BeadChip system for association with early-onset T2DM revealed novel losses in the subtelomeric regions of both 16q24.2-3 and 22q13.31-33. Subsequent high-density custom-made oligonucleotide tiling microarray analysis verified copy number losses in these regions. Furthermore, taken together with the results of our previous study [32], simultaneous copy number losses in three (4p16.3, 16q24.2-3 and 22q13.31-33) subtelomeric regions were detected in 11 of 100 T2DM patients, while none of the 100 controls had copy number losses in all three subtelomeric regions (Figures 4). This result suggests that simultaneous copy number losses in these three subtelomeric regions constitute a potentially novel and useful strategy, with high accuracy and selectivity, for predicting susceptibility to early-onset T2DM.

The CNV regions found in 4p16.3, 16q24.2-3 and 22q13.31-33 contain a number of genes (Figure 3) which might be involved in T2DM development. For example, genes involved in the glucose-induced insulin secretion cascade of pancreatic beta-cells, such as ATP5I (ATP synthase, H+ transporting, mitochondrial F0 complex, subunit E) [38], CPLX1 (complexin 1) [20] and GAK (cyclin G associated kinase) in association with CDK5 (cyclin-dependent protein kinases 5), are located in 4p16.3 [39], [40]. PPARα (peroxisome proliferator-activated receptor alpha) located in 22q13.31-33 is reportedly involved in T2DM onset and progression [32], [41]. Therefore, there is a possibility that these and other as yet unknown genes are responsible for T2DM pathogenesis through their impairments caused by copy number losses. However, these genes were not necessarily all deleted in the 11 patients who had copy number losses in all three subtelomeric regions (Figure 2). In addition, we also discovered marked accumulation of CNVs in the same subjects with T2DM, as compared with non-diabetic controls. In particular, 11 of our T2DM subjects harbored all three subtelomeric copy number losses, whereas none of the controls did. These results suggest that copy number losses are not a direct cause of T2DM development. Instead, copy number losses per se appear to have been induced by another major mechanism(s) which is also involved in T2DM development. Therefore, a putative cause for CNVs, rather than a single gene defect in the CNV regions, is involved in the onset and progression of T2DM.

As shown in Table 1, the patients with all three copy number losses did not exhibit specific clinical characteristics of T2DM. Body weights, insulin secretion and insulin therapy ratios did not differ from those of other early-onset T2DM patients. Therefore, as yet, we cannot distinguish these patients with CNVs based on their clinical findings. In other words, these patients may be identified among the entire population of early-onset T2DM patients, and possibly even in the late-onset T2DM population as well. More studies are needed to elucidate the rate of CNVs in T2DM subjects, in general, by examining these CNVs in larger populations. Since DNA samples were collected from individuals living in northeastern Japan, local factors should be taken into consideration. However, there are no apparent locality differences between our T2DM subjects and the non-diabetic controls. In addition, neither common nor other CNVs tended to be shared between the patient and control groups (data not shown). These results collectively indicate the combined CNVs to be strongly associated with T2DM, at least in subjects living in northeastern Japan. Considering that genomes uniquely correlating with T2DM have been found in East Asian countries like Japan, Korea and China [30], the CNVs in these three regions might be related to racial inherited factors. In addition, because of the small sample size, we cannot rule out the possibility that the contribution of CNVs to T2DM development might be overestimated. Further intensive studies with a larger population are needed to clarify these issues, including generality, local factors and pathogenesis.

In conclusion, copy number losses within multiple subtelomeric regions are strongly associated with early-onset T2DM. Examining the CNVs in three regions may be a useful strategy for predicting T2DM as well as possibly providing insights into its pathogenesis.

## References

[1] Poulsen P, Kyvik KO, Vaag A, Beck-Nielsen H (1999) Heritability of type II (non-insulin-dependent) diabetes mellitus and abnormal glucose tolerance–a population-based twin study. Diabetologia 42: 139–145. 10064092 10.1007/s001250051131

[2] Kaprio J, Tuomilehto J, Koskenvuo M, Romanov K, Reunanen A, et al (1992) Concordance for type 1 (insulin-dependent) and type 2 (non-insulin-dependent) diabetes mellitus in a population-based cohort of twins in Finland. Diabetologia 35: 1060–1067. 1473616 10.1007/BF02221682

[3] Meigs JB, Cupples LA, Wilson PW (2000) Parental transmission of type 2 diabetes: the Framingham Offspring Study. Diabetes 49: 2201–2207. 11118026 10.2337/diabetes.49.12.2201

[4] Weijnen CF, Rich SS, Meigs JB, Krolewski AS, Warram JH (2002) Risk of diabetes in siblings of index cases with Type 2 diabetes: implications for genetic studies. Diabet Med 19: 41–50. 11869302 10.1046/j.1464-5491.2002.00624.x

[5] Frayling TM, Evans JC, Bulman MP, Pearson E, Allen L, et al (2001) beta-cell genes and diabetes: molecular and clinical characterization of mutations in transcription factors. Diabetes 50 Suppl 1: S94–100. 11272211 10.2337/diabetes.50.2007.s94

[6] Katagiri H, Asano T, Ishihara H, Inukai K, Anai M, et al (1992) Nonsense mutation of glucokinase gene in late-onset non-insulin-dependent diabetes mellitus. Lancet 340: 1316–1317. 1360036 10.1016/0140-6736(92)92494-z

[7] van den Ouweland JM, Lemkes HH, Ruitenbeek W, Sandkuijl LA, de Vijlder MF, et al (1992) Mutation in mitochondrial tRNA(Leu)(UUR) gene in a large pedigree with maternally transmitted type II diabetes mellitus and deafness. Nat Genet 1: 368–371. 1284550 10.1038/ng0892-368

[8] Oka Y, Katagiri H, Yazaki Y, Murase T, Kobayashi T (1993) Mitochondrial gene mutation in islet-cell-antibody-positive patients who were initially non-insulin-dependent diabetics. Lancet 342: 527–528. 8102670 10.1016/0140-6736(93)91649-7

[9] Inoue H, Tanizawa Y, Wasson J, Behn P, Kalidas K, et al (1998) A gene encoding a transmembrane protein is mutated in patients with diabetes mellitus and optic atrophy (Wolfram syndrome). Nat Genet 20: 143–148. 9771706 10.1038/2441

[10] Grant SF, Thorleifsson G, Reynisdottir I, Benediktsson R, Manolescu A, et al (2006) Variant of transcription factor 7-like 2 (TCF7L2) gene confers risk of type 2 diabetes. Nat Genet 38: 320–323. 16415884 10.1038/ng1732

[11] Sakai K, Imamura M, Tanaka Y, Iwata M, Hirose H, et al (2013) Replication Study for the Association of 9 East Asian GWAS-Derived Loci with Susceptibility to Type 2 Diabetes in a Japanese Population. PLoS One 8: e76317. 24086726 10.1371/journal.pone.0076317 PMC3783369

[12] Cho YS, Chen CH, Hu C, Long J, Ong RT, et al (2012) Meta-analysis of genome-wide association studies identifies eight new loci for type 2 diabetes in east Asians. Nat Genet 44: 67–72. 10.1038/ng.1019 PMC3582398 22158537

[13] Unoki H, Takahashi A, Kawaguchi T, Hara K, Horikoshi M, et al (2008) SNPs in KCNQ1 are associated with susceptibility to type 2 diabetes in East Asian and European populations. Nat Genet 40: 1098–1102. 18711366 10.1038/ng.208

[14] Yasuda K, Miyake K, Horikawa Y, Hara K, Osawa H, et al (2008) Variants in KCNQ1 are associated with susceptibility to type 2 diabetes mellitus. Nat Genet 40: 1092–1097. 18711367 10.1038/ng.207

[15] Scott LJ, Mohlke KL, Bonnycastle LL, Willer CJ, Li Y, et al (2007) A genome-wide association study of type 2 diabetes in Finns detects multiple susceptibility variants. Science 316: 1341–1345. 17463248 10.1126/science.1142382 PMC3214617

[16] Sladek R, Rocheleau G, Rung J, Dina C, Shen L, et al (2007) A genome-wide association study identifies novel risk loci for type 2 diabetes. Nature 445: 881–885. 17293876 10.1038/nature05616

[17] Stranger BE, Forrest MS, Dunning M, Ingle CE, Beazley C, et al (2007) Relative impact of nucleotide and copy number variation on gene expression phenotypes. Science 315: 848–853. 17289997 10.1126/science.1136678 PMC2665772

[18] Gonzalez E, Kulkarni H, Bolivar H, Mangano A, Sanchez R, et al (2005) The influence of CCL3L1 gene-containing segmental duplications on HIV-1/AIDS susceptibility. Science 307: 1434–1440. 15637236 10.1126/science.1101160

[19] Hayakawa T, Noda M, Yasuda K, Yorifuji H, Taniguchi S, et al (1998) Ethidium bromide-induced inhibition of mitochondrial gene transcription suppresses glucose-stimulated insulin release in the mouse pancreatic beta-cell line betaHC9. J Biol Chem 273: 20300–20307. 9685380 10.1074/jbc.273.32.20300

[20] Abderrahmani A, Niederhauser G, Plaisance V, Roehrich ME, Lenain V, et al (2004) Complexin I regulates glucose-induced secretion in pancreatic beta-cells. J Cell Sci 117: 2239–2247. 15126625 10.1242/jcs.01041

[21] Redon R, Ishikawa S, Fitch KR, Feuk L, Perry GH, et al (2006) Global variation in copy number in the human genome. Nature 444: 444–454. 17122850 10.1038/nature05329 PMC2669898

[22] Stefansson H, Rujescu D, Cichon S, Pietilainen OP, Ingason A, et al (2008) Large recurrent microdeletions associated with schizophrenia. Nature 455: 232–236. 18668039 10.1038/nature07229 PMC2687075

[23] Rare chromosomal deletions and duplications increase risk of schizophrenia. Nature 455: 237–241. 18668038 10.1038/nature07239 PMC3912847

[24] Miller DT, Shen Y, Weiss LA, Korn J, Anselm I, et al (2009) Microdeletion/duplication at 15q13.2q13.3 among individuals with features of autism and other neuropsychiatric disorders. J Med Genet 46: 242–248. 18805830 10.1136/jmg.2008.059907 PMC4090085

[25] Marshall CR, Noor A, Vincent JB, Lionel AC, Feuk L, et al (2008) Structural variation of chromosomes in autism spectrum disorder. Am J Hum Genet 82: 477–488. 18252227 10.1016/j.ajhg.2007.12.009 PMC2426913

[26] Weiss LA, Shen Y, Korn JM, Arking DE, Miller DT, et al (2008) Association between microdeletion and microduplication at 16p11.2 and autism. N Engl J Med 358: 667–675. 18184952 10.1056/NEJMoa075974

[27] Pinto D, Pagnamenta AT, Klei L, Anney R, Merico D, et al (2010) Functional impact of global rare copy number variation in autism spectrum disorders. Nature 466: 368–372. 20531469 10.1038/nature09146 PMC3021798

[28] Zhang D, Cheng L, Qian Y, Alliey-Rodriguez N, Kelsoe JR, et al (2009) Singleton deletions throughout the genome increase risk of bipolar disorder. Mol Psychiatry 14: 376–380. 19114987 10.1038/mp.2008.144 PMC2735188

[29] Conrad DF, Pinto D, Redon R, Feuk L, Gokcumen O, et al (2010) Origins and functional impact of copy number variation in the human genome. Nature 464: 704–712. 19812545 10.1038/nature08516 PMC3330748

[30] Park H, Kim JI, Ju YS, Gokcumen O, Mills RE, et al (2010) Discovery of common Asian copy number variants using integrated high-resolution array CGH and massively parallel DNA sequencing. Nat Genet 42: 400–405. 20364138 10.1038/ng.555 PMC3329635

[31] Craddock N, Hurles ME, Cardin N, Pearson RD, Plagnol V, et al (2010) Genome-wide association study of CNVs in 16,000 cases of eight common diseases and 3,000 shared controls. Nature 464: 713–720. 20360734 10.1038/nature08979 PMC2892339

[32] Kudo H, Emi M, Ishigaki Y, Tsunoda U, Hinokio Y, et al (2011) Frequent loss of genome gap region in 4p16.3 subtelomere in early-onset type 2 diabetes mellitus. Exp Diabetes Res 2011: 498460. 21754918 10.1155/2011/498460 PMC3132460

[33] Barrett MT, Scheffer A, Ben-Dor A, Sampas N, Lipson D, et al (2004) Comparative genomic hybridization using oligonucleotide microarrays and total genomic DNA. Proc Natl Acad Sci U S A 101: 17765–17770. 15591353 10.1073/pnas.0407979101 PMC535426

[34] Perry GH, Ben-Dor A, Tsalenko A, Sampas N, Rodriguez-Revenga L, et al (2008) The fine-scale and complex architecture of human copy-number variation. Am J Hum Genet 82: 685–695. 18304495 10.1016/j.ajhg.2007.12.010 PMC2661628

[35] de Smith AJ, Tsalenko A, Sampas N, Scheffer A, Yamada NA, et al (2007) Array CGH analysis of copy number variation identifies 1284 new genes variant in healthy white males: implications for association studies of complex diseases. Hum Mol Genet 16: 2783–2794. 17666407 10.1093/hmg/ddm208

[36] Sharp AJ, Hansen S, Selzer RR, Cheng Z, Regan R, et al (2006) Discovery of previously unidentified genomic disorders from the duplication architecture of the human genome. Nat Genet 38: 1038–1042. 16906162 10.1038/ng1862

[37] Lupski JR (2009) Genomic disorders ten years on. Genome Med 1: 42. 19439022 10.1186/gm42 PMC2684663

[38] Swartz DA, Park EI, Visek WJ, Kaput J (1996) The e subunit gene of murine F1F0-ATP synthase. Genomic sequence, chromosomal mapping, and diet regulation. J Biol Chem 271: 20942–20948. 8702853 10.1074/jbc.271.34.20942

[39] Kimura SH, Tsuruga H, Yabuta N, Endo Y, Nojima H (1997) Structure, expression, and chromosomal localization of human GAK. Genomics 44: 179–187. 9299234 10.1006/geno.1997.4873

[40] Wei FY, Nagashima K, Ohshima T, Saheki Y, Lu YF, et al (2005) Cdk5-dependent regulation of glucose-stimulated insulin secretion. Nat Med 11: 1104–1108. 16155576 10.1038/nm1299

[41] Flavell DM, Ireland H, Stephens JW, Hawe E, Acharya J, et al (2005) Peroxisome proliferator-activated receptor alpha gene variation influences age of onset and progression of type 2 diabetes. Diabetes 54: 582–586. 15677519 10.2337/diabetes.54.2.582

